# Emotion Classification from Electroencephalographic Signals Using Machine Learning

**DOI:** 10.3390/brainsci14121211

**Published:** 2024-11-29

**Authors:** Jesus Arturo Mendivil Sauceda, Bogart Yail Marquez, José Jaime Esqueda Elizondo

**Affiliations:** 1 Tecnológico Nacional de México, Campus Tijuana. Calz del Tecnológico 12950, Tomas Aquino, Tijuana 22414, Mexico; bogart@tectijuana.edu.mx; 2 Facultad de Ciencias Químicas e Ingeniería, Universidad Autónoma de Baja California, Calzada Universidad 14418, Parque Industrial Internacional, Tijuana 22390, Mexico; jjesqueda@uabc.edu.mx

**Keywords:** machine learning, artificial intelligence, EEG, emotion recognition, neural networks, deep learning, ShallowFBCSPNet, Deep4Net, EEGNetv4

## Abstract

Background: Emotions significantly influence decision-making, social interactions, and medical outcomes. Leveraging emotion recognition through Electroencephalography (EEG) signals offers potential advancements in personalized medicine, adaptive technologies, and mental health diagnostics. This study aimed to evaluate the performance of three neural network architectures—ShallowFBCSPNet, Deep4Net, and EEGNetv4—for emotion classification using the SEED-V dataset. Methods: The SEED-V dataset comprises EEG recordings from 16 individuals exposed to 15 emotion-eliciting video clips per session, targeting happiness, sadness, disgust, neutrality, and fear. EEG data were preprocessed with a bandpass filter, segmented by emotional episodes, and split into training (80%) and testing (20%) sets. Three neural networks were trained and evaluated to classify emotions from the EEG signals. Results: ShallowFBCSPNet achieved the highest accuracy at 39.13%, followed by Deep4Net (38.26%) and EEGNetv4 (25.22%). However, significant misclassification issues were observed, such as EEGNetv4 predicting all instances as “Disgust” or “Neutral” depending on the configuration. Compared to state-of-the-art methods, such as ResNet18 combined with differential entropy, which achieved 95.61% accuracy on the same dataset, the tested models demonstrated substantial limitations. Conclusions: Our results highlight the challenges of generalizing across emotional states using raw EEG signals, emphasizing the need for advanced preprocessing and feature-extraction techniques. Despite these limitations, this study provides valuable insights into the potential and constraints of neural networks for EEG-based emotion recognition, paving the way for future advancements in the field.

## 1. Introduction

The nervous system is a complex and intricate network that governs the functions and behaviors of living organisms. It is divided into two primary subsystems: the central nervous system (CNS) and the peripheral nervous system (PNS). The CNS, comprising the brain and spinal cord, is the control center responsible for processing sensory information, initiating responses, and maintaining cognitive functions, such as thought, memory, and emotion [1]. As the CNS’s principal organ, the brain orchestrates reactions to external stimuli, facilitates communication, and regulates emotions. It influences how humans perceive, express, and regulate emotional experiences [2].

### 1.1. Theories of Emotion

Emotions have been a focal point in psychology and neuroscience for decades, with several influential theories proposed to explain their origins, expressions, and functions. One of the most prominent is the theory of basic emotions, proposed by Paul Ekman, which posits that certain emotions, such as anger, fear, sadness, happiness, and disgust, are universal across human cultures and are expressed through innate facial expressions [3,4]. These emotions are thought to have evolved as adaptive responses to environmental challenges and opportunities, helping individuals survive by responding appropriately to stimuli [5,6].

Building upon this, Plutchik developed his “Wheel of Emotions” model, which organizes emotions into primary, secondary, and tertiary levels. His theory suggests that emotions are experienced in varying intensities and combinations, allowing for a more nuanced understanding of human emotional experiences [7]. This model has been widely used in affective computing, where the goal is to capture and interpret the complex interplay between emotional states [8]. Another influential model, Russell’s circumplex model of affect, categorizes emotions along two dimensions: arousal (high to low energy) and valence (positive to negative), providing a framework for understanding how different emotional states are interrelated [9].

The recognition and measurement of emotions are not without challenges. Although facial expressions and vocal cues provide valuable information, they are subject to individual and cultural variations, which can lead to inaccuracies in emotion-recognition systems [10]. Studies by Yan et al. [11] showed that cultural differences play a significant role in how facial expressions are perceived and interpreted, underscoring the need for more direct and objective measures of emotional states [12,13,14]. As a result, physiological signals, such as those measured using EEG, have gained attention as a more reliable source for emotion detection [15].

### 1.2. Emotion Recognition Through EEG

Electroencephalography (EEG) is a non-invasive method used to measure electrical activity in the brain by placing electrodes on the scalp. EEG is particularly well-suited for emotion recognition because of its high temporal resolution, which allows for capturing rapid changes in brain activity in response to emotional stimuli [16,17]. Unlike other neuroimaging techniques, such as functional magnetic resonance imaging (fMRI) or positron emission tomography (PET), which have a high spatial resolution but a limited temporal resolution, EEG provides a continuous and real-time representation of brain dynamics, making it an ideal tool for real-time emotion-recognition applications [18].

EEG-based emotion recognition is built upon analyzing specific frequency bands within EEG signals. Brain waves are typically categorized into five frequency bands: delta (1–4 Hz), theta (4–8 Hz), alpha (8–13 Hz), beta (13–30 Hz), and gamma (30–50 Hz), with each being associated with different mental states and emotional responses [18,19]. For example, increases in alpha activity have been linked to relaxed states, while heightened beta activity is associated with concentration and arousal. By examining changes in these frequency bands, it is possible to infer an individual’s emotional state [20].

Despite its potential, EEG signals are prone to noise and artifacts, such as those produced by eye movements, muscle activity, or environmental interference. This necessitates applying sophisticated signal-processing techniques, such as filtering, artifact removal, and feature extraction, to clean the data and extract meaningful information [21]. Several preprocessing techniques, including Independent Component Analysis (ICA) and wavelet transforms, were proposed to improve the quality of the signals before feeding them into machine learning models for classification [22,23,24]. Removing artifacts is critical for ensuring the accuracy of emotion-recognition systems based on EEG [25,26].

### 1.3. Machine Learning and Deep Learning for EEG-Based Emotion Recognition

The emergence of machine learning (ML) and deep learning (DL) techniques has revolutionized the field of emotion recognition, particularly in the context of processing complex EEG data. Machine learning refers to developing algorithms that can learn from data and make decisions with minimal human intervention [27,28,29,30,31]. In contrast, deep learning, a subset of machine learning, leverages neural networks with multiple layers that can capture intricate patterns in data through hierarchical feature extraction [32,33,34,35,36]. DL demonstrated remarkable success in tasks that involved large and complex datasets, such as those generated by EEG signals [37].

Various deep learning models were applied to EEG-based emotion recognition, each with strengths and limitations [37]. Traditional machine learning approaches, such as Support Vector Machines (SVMs) and K-Nearest Neighbors (KNNs), were effective in some contexts but often required manual feature extraction [38]. In contrast, deep learning models, such as convolutional neural networks (CNNs), can automatically learn relevant features from raw EEG data, eliminating the need for manual feature engineering [39]. This has made CNNs a popular choice for emotion recognition from EEG signals.

The SJTU Emotion EEG Dataset (SEED), developed by the BCMI laboratory, has become an essential resource for training and testing these machine learning models. The dataset includes EEG recordings of participants exposed to emotional stimuli that elicit five emotional states: happiness, sadness, fear, disgust, and neutrality [40]. The SEED-V dataset, an extension of the original SEED, also incorporates multimodal data, such as eye movements, to further enhance the accuracy of emotion-classification systems [41].

### 1.4. Use of RAW EEG Signals

The SEED-V dataset provides both raw EEG signals and preprocessed signals derived using differential entropy (DE). For this study, we specifically chose to work with the raw EEG signals for the following reasons:

- Flexibility in feature learning: Using raw EEG signals allowed the models to directly learn meaningful features from the data rather than relying on predefined transformations like DE. This approach provided greater flexibility in identifying both time-domain and frequency-domain features relevant to emotion classification.
- Avoiding predefined feature bias: While DE is effective at capturing temporal and stochastic EEG dynamics, it imposes assumptions about the data’s structure. By working with raw signals, the models retain the ability to explore a wider range of features that may not be represented by DE.
- Baseline comparison: Employing raw signals establishes a baseline for evaluating the capacity of the selected models to process minimally preprocessed data. This enables a more direct comparison of model architectures and their performances without the influence of external feature-extraction techniques.

### 1.5. Selected Models

The models chosen for this study—ShallowFBCSPNet, Deep4Net, and EEGNetv4—were specifically selected based on their abilities to address different aspects of EEG-based emotion recognition, offering complementary strengths:

- ShallowFBCSPNet: This model was inspired by the Filter Bank Common Spatial Patterns (FBCSP), a proven approach for EEG signal analysis, particularly for extracting frequency-specific features. ShallowFBCSPNet is well-suited for tasks where emotions are linked to distinct frequency bands, such as those in the SEED-V dataset. Its shallow architecture provides interpretable insights while efficiently capturing key signal components.
- Deep4Net: Deep4Net is a deeper convolutional architecture designed to capture hierarchical and non-linear patterns within EEG data. Its depth allows it to learn more abstract representations, which are crucial for distinguishing subtle differences between emotional states. This makes it particularly effective for complex datasets like SEED-V, where some emotions (e.g., fear and disgust) share overlapping features.
- EEGNetv4: EEGNetv4 was selected for its lightweight and efficient design, specifically tailored for real-time applications. Its depthwise and separable convolutions reduce the computational complexity, making it an attractive option for resource-constrained environments. Despite its compact design, EEGNetv4 retains the capacity to process multi-channel EEG data effectively.

### 1.6. Deep Learning Architectures for EEG-Based Emotion Recognition

Three notable deep learning architectures for EEG-based emotion recognition are ShallowFBCSPNet, Deep4Net, and EEGNetv4. Each one offers distinct advantages for processing EEG signals.

**Figure 1:**
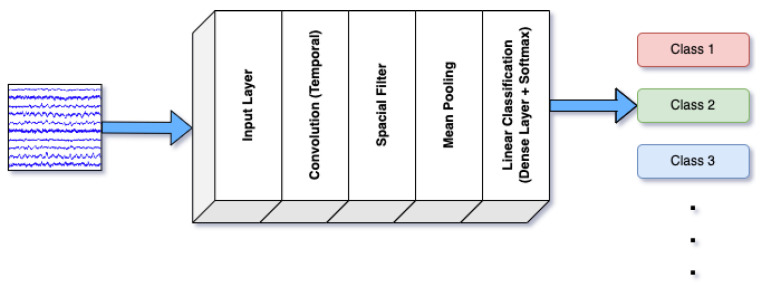
Architecture of ShallowFBCSPNet.

ShallowFBCSPNet was designed to extract band-power features from EEG signals across multiple frequency bands. It combines shallow and deep network layers to capture local and global patterns in the brain’s electrical activity, making it practical for distinguishing between various emotional states [42]. The model leverages the frequency-specific characteristics of EEG signals, which are crucial for decoding complex emotional information. Figure 1 illustrates the architecture of ShallowFBCSPNet. The arrows represent the flow of data through the network, while the colors at the output correspond to the different classes of EEG signals identified by the model, such as emotional states, movement-related signals, or other cognitive processes.

**Figure 2:**
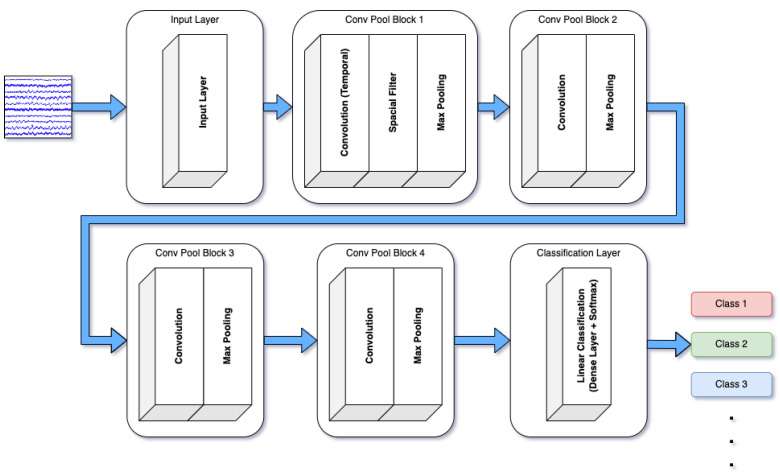
Architecture of Deep4Net.

Deep4Net is another deep learning model designed to capture hierarchical features in EEG data. This model uses multiple convolutional layers to extract abstract patterns from EEG signals, making it highly effective for classifying emotions that exhibit subtle differences in brain activity [42]. Deep4Net excels in recognizing emotions through its ability to process complex, non-linear data. The architecture of Deep4Net, shown in Figure 2, depicts its hierarchical structure, with layers progressively extracting more abstract features from EEG data. Arrows indicate data flow between layers, while the colors at the output represent the various EEG signal classes predicted by the model, such as emotional states, or mental tasks.

**Figure 3:**
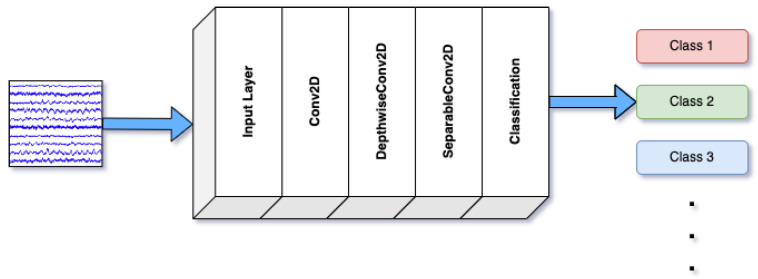
Architecture of EEGNetv4.

EEGNetv4 is a lightweight and efficient model tailored for real-time brain-computer interface (BCI) applications. Its architecture includes depthwise and separable convolutions, which significantly reduce the number of parameters while maintaining the model’s accuracy in detecting emotional states [43]. EEGNetv4 is especially advantageous for portable and low-power applications with limited computational resources. Figure 3 demonstrates the design of EEGNetv4. The arrows highlight the directional data flow, and the colors at the output layer correspond to the different classes of EEG signals identified by the model, including signals associated with movement, thinking, or emotional states.

## 2. Materials and Methods

### 2.1. Dataset

**Figure 4:**
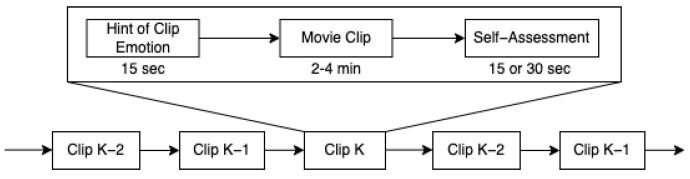
SEED-V experimental process.

We used the SEED dataset; this dataset has several versions. The first version, SEED, detected three positive, negative, and neutral mental states using EEG and eye movement data. In this study, we used the SEED-V version, which contains data for five mental states, happy, sad, disgust, neutral, and fear, using EEG and eye movement data. To ensure the stability of emotion recognition, each participant underwent the experiment three times, with a minimum interval of three days between sessions. In each session, the participant needed to watch 15 stimulating audiovisual materials, with 3 for each type of emotion. The participants viewed one of the film clips while their EEG signals and eye movements were recorded using a 62-channel ESI NeuroScan System and SMI eye-tracking glasses. To prevent participants from becoming bored, different materials were shown each time. Each session lasted approximately 50 min in total. Before each stimulus was played, there was a 15 s introduction to provide background on the material and the emotions it aimed to evoke. After viewing the stimuli, participants were given 15 or 30 s for self-assessment and rest, depending on the type of material. For stimuli designed to evoke disgust or fear, the rest period was 30 s, while for happiness, neutrality, and sadness, the rest period was 15 s. During the self-assessment phase, participants were asked to rate the effectiveness of the stimuli in inducing the intended emotion using a scale from 0 to 5, where 5 represented the most potent effect and 0 represented no effect. For example, if a participant felt joy after watching a clip intended to induce happiness, they were to rate it 4 or 5; if they felt nothing, they were to rate it 0. For neutral stimuli, if the participant’s mood fluctuated, they were instructed to score 0, while a natural, steady mood was rated 5. This process is shown in Figure 4.

### 2.2. Hardware and Software

The hardware we used in this study had the following specifications: Google Colab notebook with a T4 GPU as a processing unit; the hardware used to create the dataset was a 62-channel ESI NeuroScan System and SMI eye-tracking glasses. The software used for reading the EEG signals, preprocessing, analysis, feature extraction, and preparation of the dataset was Python version 3.10.12, along with an EEG-processing library called MNE-Python [44], which is an open-source Python package for exploring, visualizing, and analyzing human neurophysiological data, such as MEG, EEG, sEEG, and ECoG. It includes data input/output modules, preprocessing, visualization, source estimation, time-frequency analysis, connectivity analysis, machine learning, and statistics.

### 2.3. Input Data

SEED-V provided the EEG RAW data and also pre-processed data; the pre-processed data were downsampled to a 200 Hz sampling rate, and the noise was removed using a bandpass filter that ranged from 1 Hz to 75 Hz, followed by the application of differential entropy (DE) within each segment across five frequency bands: (1) delta: 1–4 Hz, (2) theta: 4–8 Hz, (3) alpha: 8–14 Hz, (4) beta: 14–31 Hz, and (5) gamma: 31–50 Hz. We used the RAW EEG signals, where were applied the bandpass filter between 1 Hz and 75 Hz to filter the noise and remove the artifacts. After this, since the RAW EEG signal was provided as only one file for each person by session, we had 16 people, with data from 3 sessions, for 48 files in total, we needed to apply data segmentation for each emotion by applying a label in the time range for each of the videos watched. Therefore, we divided each file into the 15 videos watched to provide a total of 720 files labeled with the emotion that was being evoked; the RAW EEG naming convention was personNumber_sessionNumber_Date, for example 1_1_20180804, while the naming convention after the segmentation was personNumber_sessionNumber_videoNumber_emotion, for example 1_1_11_happy.

### 2.4. Proposed Methodology for SEED-V EEG Signal Processing

**Figure 5:**
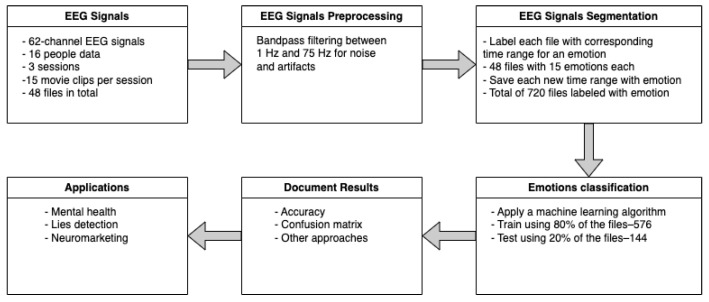
Proposed methodology.

The used methodology is shown in Figure 5 below, where each block is described in the following subsections.

### 2.5. EEG Signal Channels

**Figure 6:**
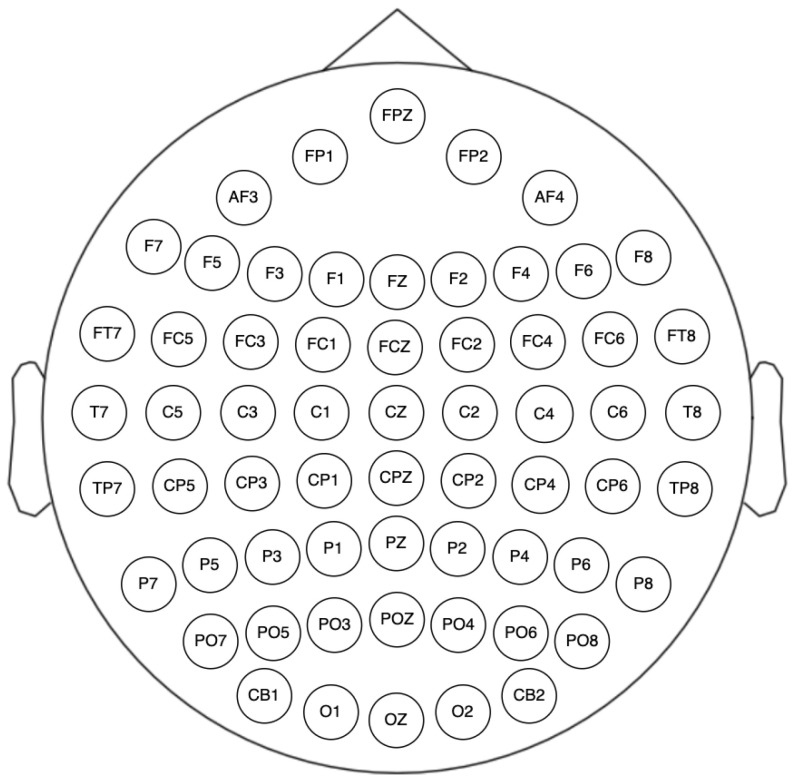
The 62-channel ESI NeuroScan sensors placement.

The headband used in the dataset was a 62-channel ESI NeuroScan System. The sensors’ placement is shown in Figure 6. Channels M1, M2, VEO, and HEO were discarded since M1 and M2 were ground electrodes, and VEO and HEO were used for eye movement activity.

### 2.6. EEG Signals Processing

**Figure 7:**
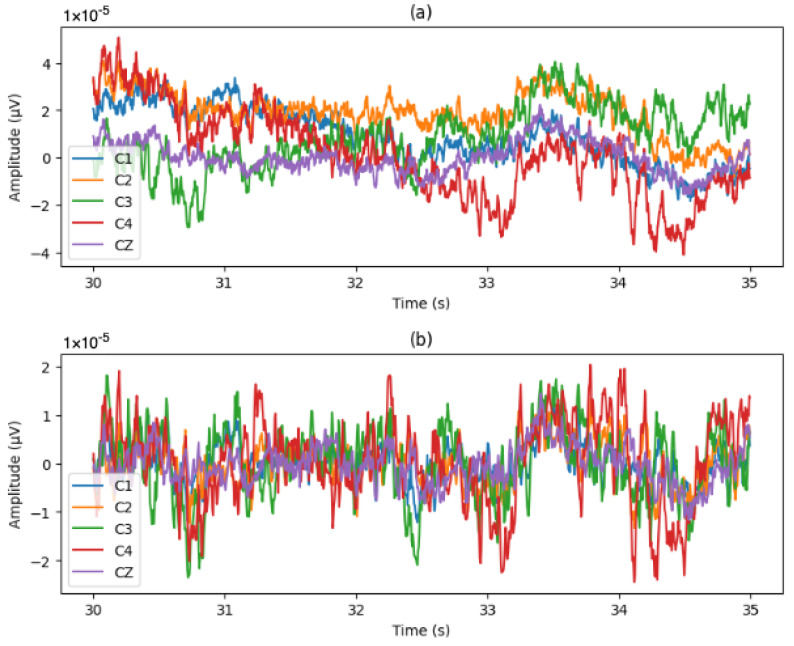
EEG signals from electrodes C3, C1, CZ, C2, and C4. The first 5 s of clip 1 from session 1, subject 1 is displayed. (**a**) RAW EEG signal and (**b**) processed EEG signal.

**Figure 8:**
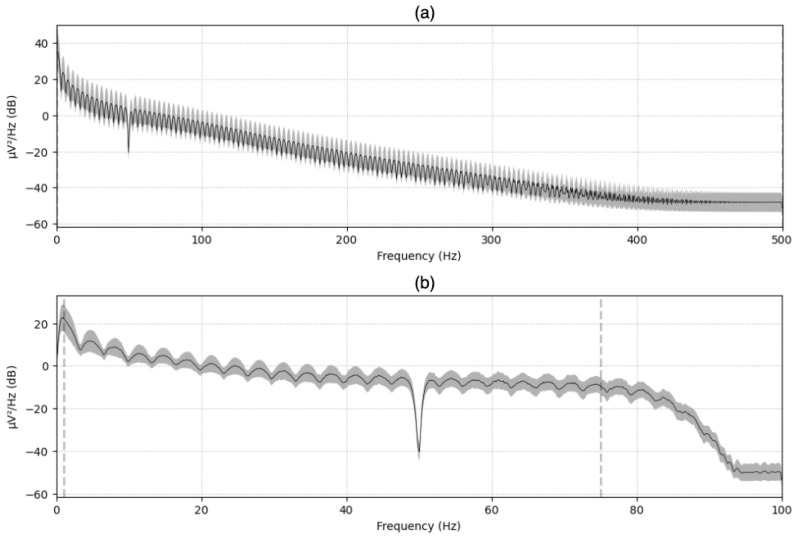
Power Spectral Density plot corresponding to clip #1 from session 1, subject 1. (**a**) RAW EEG signals PSD and (**b**) processed EEG signals PSD.

Since we used the RAW signals from the dataset, we needed to apply downsampling from 1000 Hz to 200 Hz signals to reduce the load when processing the data, and we applied a bandpass filter from 1 Hz to 75 Hz for the noise and artifacts. Figure 7 shows the EEG signals from subject 1, session 1, and clip 1 before and after applying downsampling and filtering. Figure 8 shows the Power Spectral Density (PSD) before and after the processing.

### 2.7. EEG Signals Segmentation

**Figure 9:**
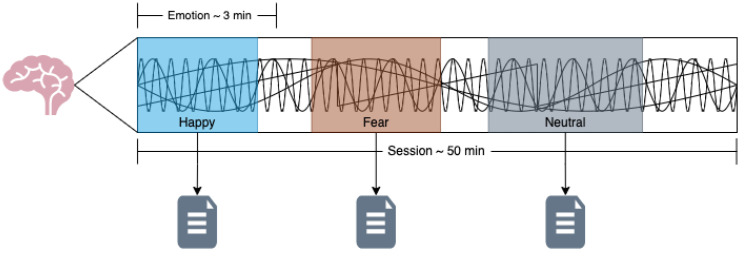
Signal segmentation process to save a new file per emotion.

Since the dataset provides one file per person per session, we needed to separate each session into individual emotions. We had the time ranges corresponding to each emotion clip in each session, and there were 15 emotion clips per session, so for this purpose, we labeled the whole session by the time range and ended up having 15 files per person per session. This made it easier to process since we already had a file corresponding to one concrete emotion that we could use to feed our model. The overall process is shown in Figure 9.

### 2.8. Machine Learning Models for Emotion Classification

This study evaluated three machine learning models to classify emotions based on the preprocessed EEG signals: ShallowFBCSPNet, Deep4Net, and EEGNetv4. ShallowFBCSPNet: This model, introduced by Schirrmeister et al. [42], was designed to extract band-power features across multiple frequency bands, which is particularly relevant for EEG analysis. The model is structured to capture both shallow and deep patterns in EEG signals, making it suitable for decoding complex emotional states. The architecture of ShallowFBCSPNet is detailed in Table 1, which outlines its layers, output shapes, and parameter counts.

Deep4Net: Deep4Net is a deeper convolutional neural network architecture developed to capture hierarchical features in EEG data. This model, also introduced by Schirrmeister et al. [42], was specifically tailored to detect more abstract patterns in the data, which are crucial for distinguishing between similar emotional states. The architecture of Deep4Net is detailed in Table 2, which outlines its layers, output shapes, and parameter counts.

EEGNetv4: EEGNetv4, developed by Lawhern et al. [43], is a compact and efficient network architecture designed for EEG-based brain-computer interface (BCI) applications. The model employs depthwise and separable convolutions, making it lightweight while retaining the ability to capture both spatial and temporal features in EEG signals. Table 3 provides a detailed breakdown of EEGNetv4’s architecture, showing the layers, output shapes, and parameter counts.

Each model was trained and evaluated on the SEED-V dataset, with 80% of the data used for training and 20% reserved for testing. The performance of each model was assessed in terms of accuracy, precision, and recall, with the results detailed in the subsequent sections.

### 2.9. Hyperparameter Selection Process

The hyperparameters for each model were optimized using a systematic grid search combined with cross-validation. The following strategy was employed:

- Learning rate: explored the values 0.0001, 0.001, 0.01, and 0.1 to balance the convergence speed and stability during training.
- Batch size: 4, 6, and 8 values were tested to accommodate the computational constraints while ensuring sufficient gradient updates.
- Weight decay: regularization was applied with values that ranged from 0.05 to 0.1 to mitigate overfitting.
- Epochs: these were fixed at 100, 120, or 200, depending on the architecture’s capacity to learn effectively within a reasonable timeframe.

Each model’s hyperparameters were fine-tuned based on validation loss minimization to enhance the generalization while reducing overfitting.

### 2.10. Source Code

The source code used for preprocessing, training, and evaluating the models is publicly available in our GitHub repository (https://github.com/artmen1516/eeg-emotion-classification-seedv, accessed on 20 September 2024) [45].

## 3. Results

This section presents the performances of three machine learning models—EEGNetv4, Deep4Net, and ShallowFBCSPNet—evaluated across different hyperparameter configurations. ShallowFBCSPNet achieved the highest accuracy (39.13%) under optimized configurations, while EEGNetv4 demonstrated a lower performance despite its compact design, indicating limitations in its ability to generalize. The results are discussed in terms of accuracy, misclassification rates, and the ability of each model to generalize across different emotional states. Visual representations, including confusion matrices and loss curves, further support the analysis results to provide a comprehensive understanding of the models’ behaviors.

### 3.1. Performance of EEGNetv4

The EEGNetv4 model, designed for efficiency and real-time applications, demonstrated consistent yet modest performances across the tested configurations. The following subsections describe the results of the two configurations evaluated.

#### 3.1.1. Configuration 1

**Figure 10:**
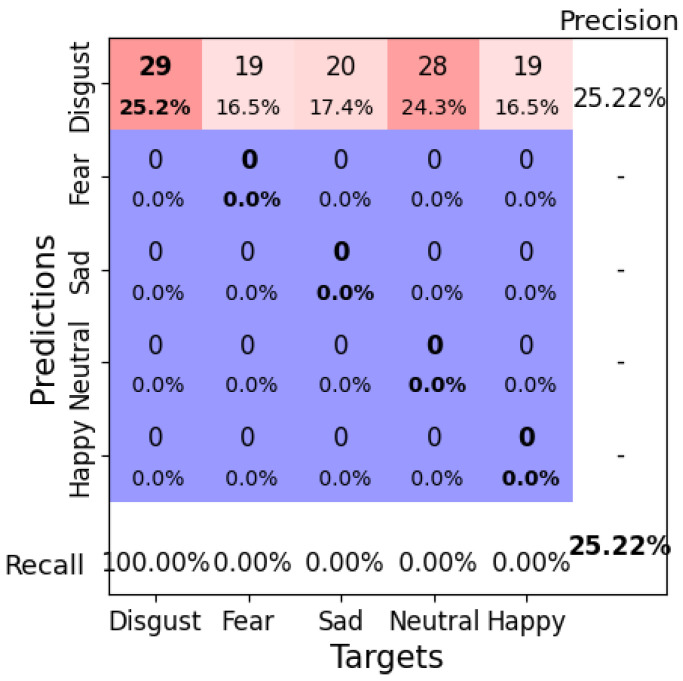
Confusion matrix for EEGNetv4 configuration 1 (learning rate = 0.001, weight decay = 0.1, batch size = 8, epochs = 100). Bold values along the diagonal represent correctly classified instances where the predicted emotion matches the target emotion. The bold percentage in the bottom-right corner indicates the overall accuracy of the model. The heatmap color scheme highlights frequencies, with darker red shades representing higher frequencies and darker purple shades indicating lower frequencies.

**Figure 11:**
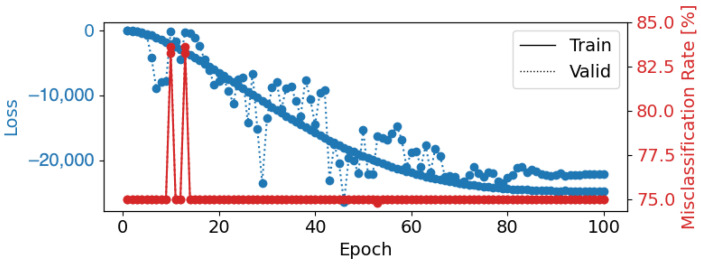
Epoch loss and misclassification rate for EEGNetv4 Configuration 1 (learning rate = 0.001, weight decay = 0.1, batch size = 8, epochs = 100). The blue lines represent training (solid) and validation (dotted) loss, while the red line shows the misclassification rate.

Using a learning rate of 0.001, a weight decay of 0.1, a batch size of 8, and 100 epochs, EEGNetv4 achieved a test accuracy of 25.22%. However, as shown in the confusion matrix (Figure 10), the model exhibited severe misclassification issues: all instances across the five emotional categories were predicted as “Disgust”. This indicates that the model failed to generalize and heavily biased its predictions toward one class (Figure 10). The corresponding loss and misclassification rate over the epochs are shown in Figure 11, which indicates that the model may have reached a plateau early, limiting further improvements.

#### 3.1.2. Configuration 2

**Figure 12:**
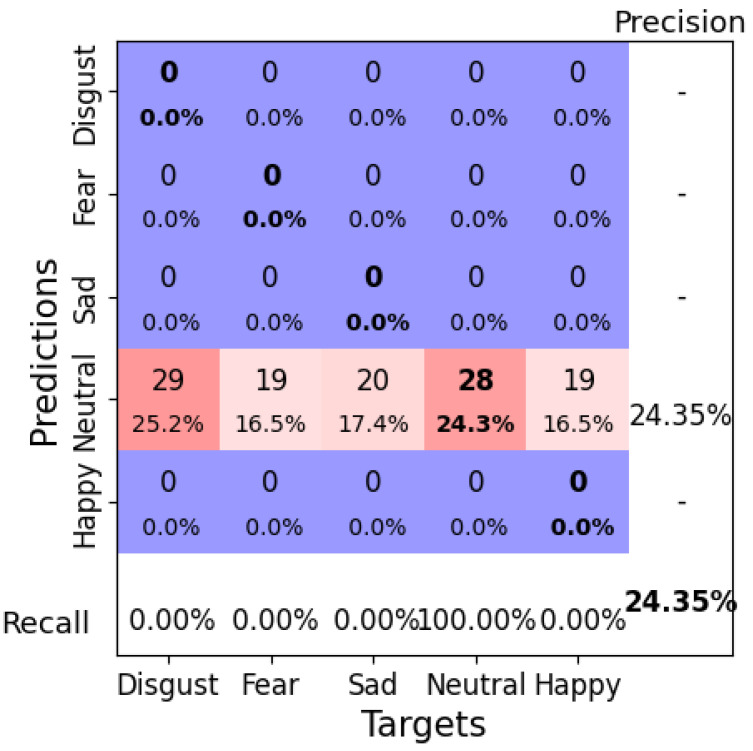
Confusion matrix for EEGNetv4 Configuration 2 (learning rate = 0.01, weight decay = 0.05, batch size = 6, epochs = 100). Bold values along the diagonal represent correctly classified instances where the predicted emotion matches the target emotion. The bold percentage in the bottom-right corner indicates the overall accuracy of the model. The heatmap color scheme highlights frequencies, with darker red shades representing higher frequencies and darker purple shades indicating lower frequencies.

**Figure 13:**
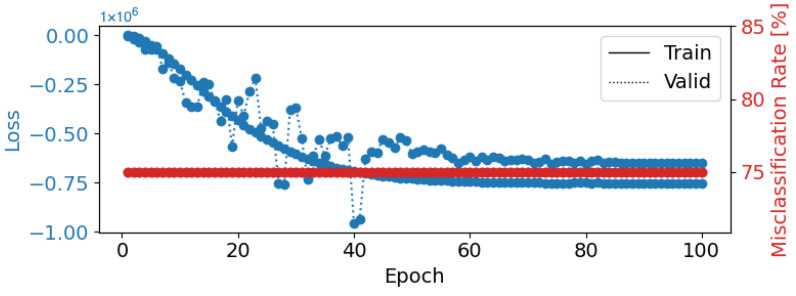
Epoch loss and misclassification rate for EEGNetv4 Configuration 2 (learning rate = 0.01, weight decay = 0.05, batch size = 6, epochs = 100). The blue lines represent training (solid) and validation (dotted) loss, while the red line shows the misclassification rate.

With a learning rate of 0.01, a weight decay of 0.05, a batch size of 6, and 100 epochs, EEGNetv4 achieved a test accuracy of 24.35%. However, as shown in the confusion matrix (Figure 12), the model classified all instances as “Neutral”, regardless of their true labels (Figure 12). This demonstrates a similar bias issue observed in Configuration 1 but with predictions skewed toward a different class. The corresponding loss and misclassification rate over the epochs are shown in Figure 13.

### 3.2. Performance of Deep4Net

Deep4Net, a deep convolutional neural network, outperformed EEGNetv4 by capturing more complex hierarchical features from the EEG data. This model showed varying results depending on the configuration used.

#### 3.2.1. Configuration 1

**Figure 14:**
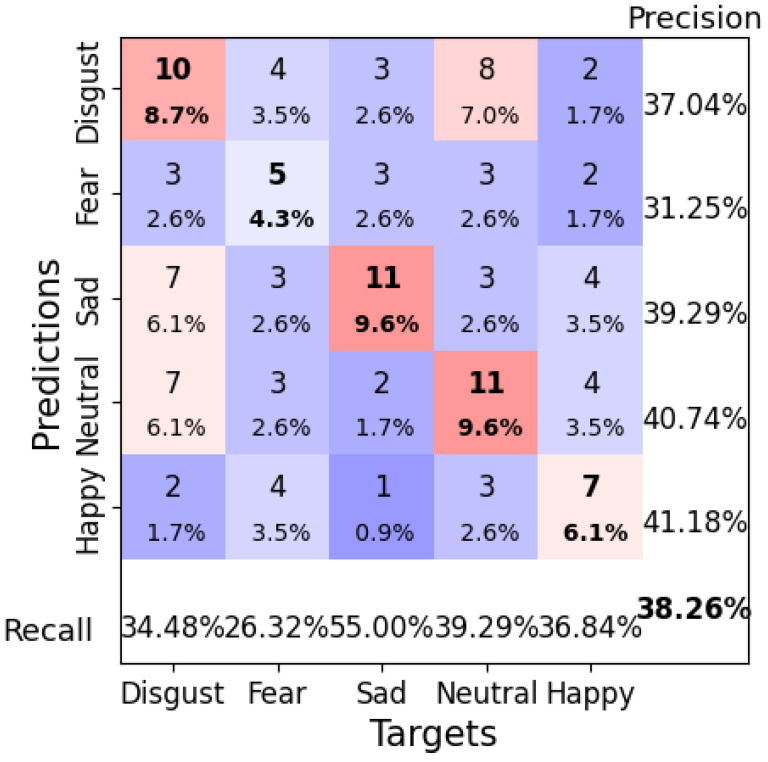
Confusion matrix for Deep4Net Configuration 1 (learning rate = 0.01, weight decay = 0.1, batch size = 8, epochs = 100). Bold values along the diagonal represent correctly classified instances where the predicted emotion matches the target emotion. The bold percentage in the bottom-right corner indicates the overall accuracy of the model. The heatmap color scheme highlights frequencies, with darker red shades representing higher frequencies and darker purple shades indicating lower frequencies.

**Figure 15:**
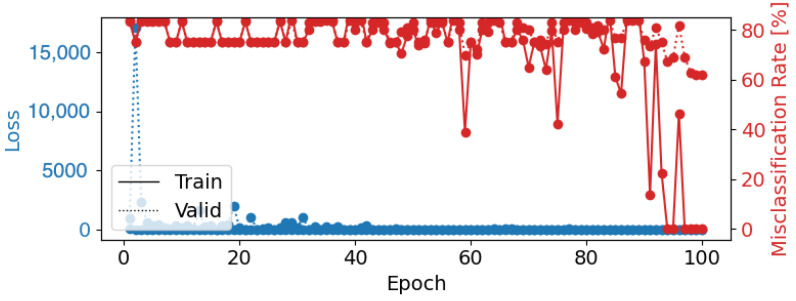
Epoch loss and misclassification rate for Deep4Net Configuration 1 (learning rate = 0.01, weight decay = 0.1, batch size = 8, epochs = 100). The blue lines represent training (solid) and validation (dotted) loss, while the red line shows the misclassification rate.

With a learning rate of 0.01, a weight decay of 0.1, a batch size of 8, and 100 epochs, Deep4Net achieved its highest test accuracy of 38.26%. The model excelled when classifying “Sad” and “Happy” emotions, as reflected in the confusion matrix (Figure 14). However, the misclassification rates remained high for other emotions, such as “Disgust” and “Fear”, indicating a need for further refinement. The loss curve in Figure 15 shows that the model may have been overfitting after a certain point, suggesting that early stopping could have improved the generalization.

#### 3.2.2. Configuration 2

**Figure 16:**
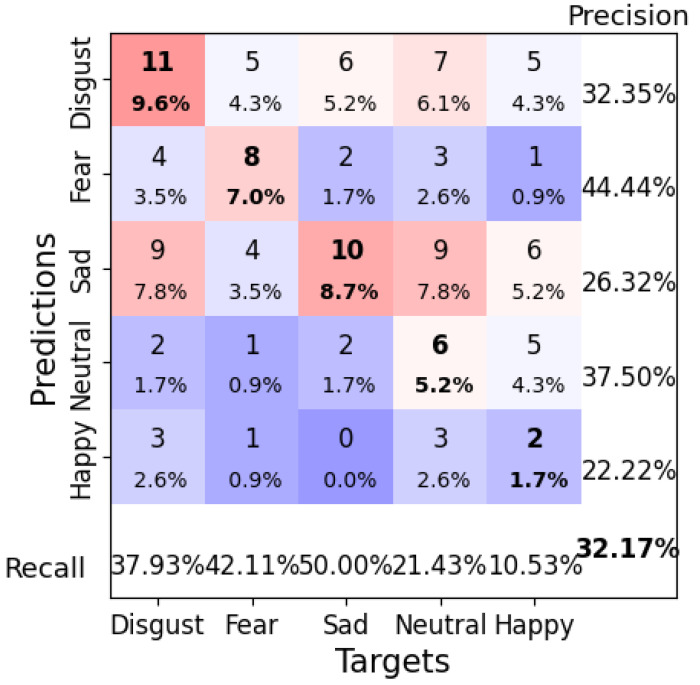
Confusion matrix for Deep4Net Configuration 2 (learning rate = 0.0005, weight decay = 0.05, batch size = 8, epochs = 100). Bold values along the diagonal represent correctly classified instances where the predicted emotion matches the target emotion. The bold percentage in the bottom-right corner indicates the overall accuracy of the model. The heatmap color scheme highlights frequencies, with darker red shades representing higher frequencies and darker purple shades indicating lower frequencies.

**Figure 17:**
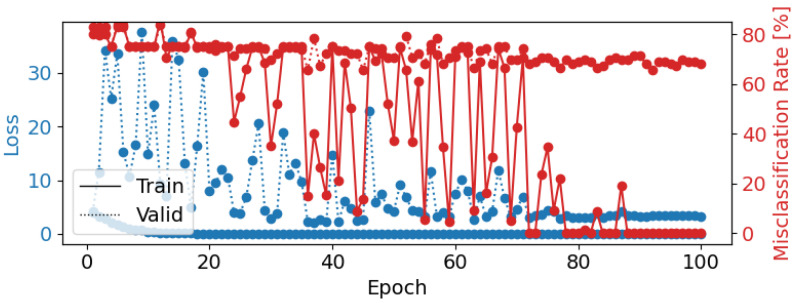
Epoch loss and misclassification rate for Deep4Net Configuration 2 (learning rate = 0.0005, weight decay = 0.05, batch size = 8, epochs = 100). The blue lines represent training (solid) and validation (dotted) loss, while the red line shows the misclassification rate.

A second configuration using a learning rate of 0.0005, a weight decay of 0.05, a batch size of 8, and 100 epochs resulted in a test accuracy of 32.17%. The model’s performance in distinguishing between similar emotional states, such as “Sad” and “Happy”, was still suboptimal (Figure 16). While this configuration provided a more stable loss curve (Figure 17), it suggests that Deep4Net’s deeper architecture, while powerful, may require more training data or advanced regularization techniques to reduce overfitting and improve the generalization.

### 3.3. Performance of ShallowFBCSPNet

ShallowFBCSPNet demonstrated the highest accuracy among the three models, showing promise for emotion-classification tasks. This model was designed to extract band-power features across multiple frequency bands, making it particularly effective for EEG data.

#### 3.3.1. Configuration 1

**Figure 18:**
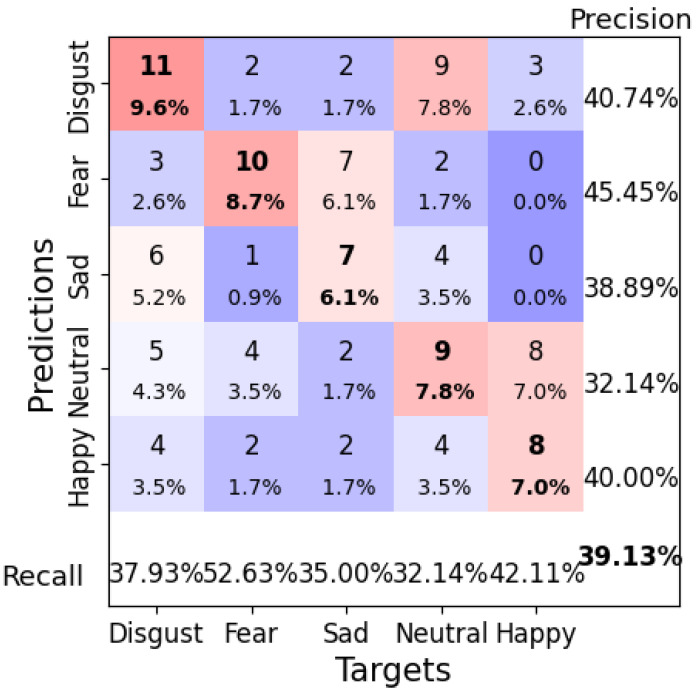
Confusion matrix for ShallowFBCSPNet Configuration 1 (learning rate = 0.0001, weight decay = 0.05, batch size = 8, epochs = 200). Bold values along the diagonal represent correctly classified instances where the predicted emotion matches the target emotion. The bold percentage in the bottom-right corner indicates the overall accuracy of the model. The heatmap color scheme highlights frequencies, with darker red shades representing higher frequencies and darker purple shades indicating lower frequencies.

**Figure 19:**
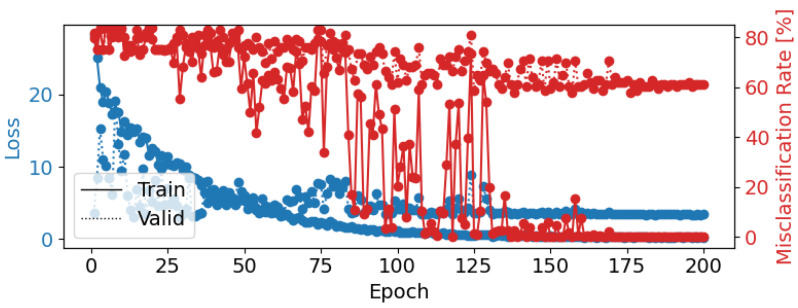
Epoch loss and misclassification rate for ShallowFBCSPNet Configuration 1 (learning rate = 0.0001, weight decay = 0.05, batch size = 8, epochs = 200). The blue lines represent training (solid) and validation (dotted) loss, while the red line shows the misclassification rate.

The optimal configuration for ShallowFBCSPNet had a learning rate of 0.0001, a weight decay of 0.05, a batch size of 8, and 200 epochs, which resulted in a test accuracy of 39.13%. The confusion matrix (Figure 18) shows that the model performed well when classifying “Fear” and “Happy” emotions. Still, the high misclassification rates for “Neutral” and “Sad” suggest that further optimization is needed. The loss curve in Figure 19 shows a stable decrease in the loss, indicating that the model learned effectively throughout the training process.

#### 3.3.2. Configuration 2

**Figure 20:**
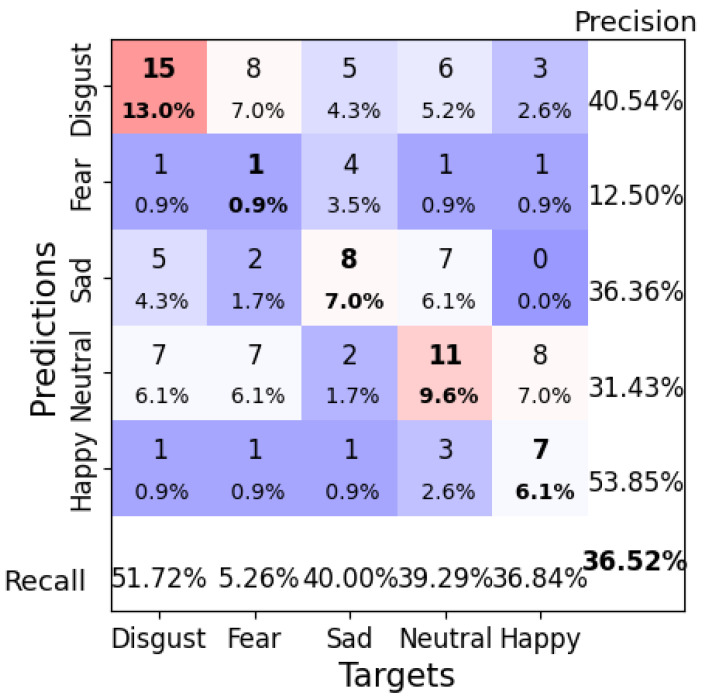
Confusion matrix for ShallowFBCSPNet Configuration 2 (learning rate = 0.005, weight decay = 0.1, batch size = 4, epochs = 120). Bold values along the diagonal represent correctly classified instances where the predicted emotion matches the target emotion. The bold percentage in the bottom-right corner indicates the overall accuracy of the model. The heatmap color scheme highlights frequencies, with darker red shades representing higher frequencies and darker purple shades indicating lower frequencies.

**Figure 21:**
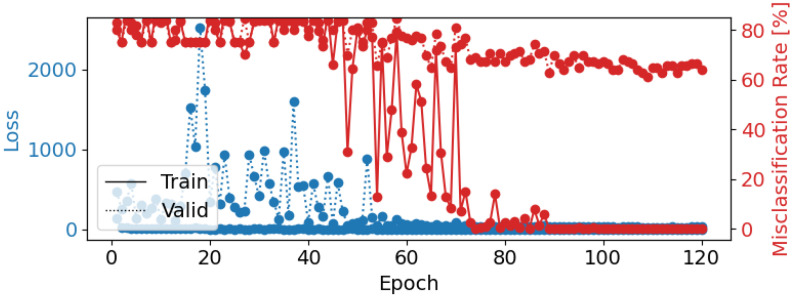
Epoch loss and misclassification rate for ShallowFBCSPNet Configuration 2 (learning rate = 0.005, weight decay = 0.1, batch size = 4, epochs = 120). The blue lines represent training (solid) and validation (dotted) loss, while the red line shows the misclassification rate.

Another effective configuration used a learning rate of 0.005, a weight decay of 0.1, a batch size of 4, and 120 epochs, which resulted in a test accuracy of 36.52%. This configuration showed improved classification for the “Happy” and “Disgust” classes but struggled with “Neutral” and “Fear” (Figure 20). The loss curve (Figure 21) indicates that the model might have benefited from a lower learning rate or additional epochs to refine the learned features further.

### 3.4. Summary of Findings

This study evaluated three neural network architectures—EEGNetv4, Deep4Net, and ShallowFBCSPNet—on the SEED-V dataset for emotion classification. ShallowFBCSPNet demonstrated the highest accuracy at 39.13%, followed by Deep4Net with 38.26%, while EEGNetv4 performed the worst, with a maximum accuracy of 25.22%. However, all models exhibited significant limitations in generalizing across the five emotional states. A summary of the models, configurations, hyperparameters, and performance metrics is provided in Table 4.

Key observations:

- EEGNetv4:
  - Configuration 1 classified all instances as “Disgust”, indicating severe overfitting.
  - Configuration 2 classified all instances as “Neutral”, reflecting underfitting and poor feature representation.
- Deep4Net:
  - Configuration 1 performed relatively well for “Sad” and “Happy” emotions but struggled with “Fear” and “Disgust”.
  - Configuration 2 exhibited better stability in training but had difficulty distinguishing between similar emotions.
- ShallowFBCSPNet:
  - Configuration 1 showed strong classification for “Disgust” and “Sad”, while it struggled with “Fear” and “Neutral”.
  - Configuration 2 improved its performance for “Happy” and “Neutral” but struggled with “Disgust” and “Fear”.

## 4. Comparative Analysis with Existing Studies

In this section, we compare the results of our study with those reported in recent studies using the SEED-V dataset for EEG-based emotion recognition. Table 5 summarizes the methodologies, key features, and performance metrics of different studies, highlighting the strengths and limitations of our approach.

### 4.1. Overview of Studies

- Our study: We evaluated three models (ShallowFBCSPNet, Deep4Net, and EEGNetv4) with bandpass filtering (1–75 Hz) and segmentation as preprocessing methods. No advanced feature-extraction techniques were used, and we achieved a maximum accuracy of 39.13% using ShallowFBCSPNet. Challenges were noted in classifying emotions like fear and disgust due to limited feature representation.
- Yao et al. (2024) [46]: Proposed a combination of ResNet18 with differential entropy (DE) for feature extraction that employed artifact rectification and a Short-Time Fourier Transform (STFT). Achieved a mean classification accuracy of 95.61%.
- Zhou et al. (2023) [47]: Used a Progressive Graph Convolution Network (PGCN) for feature learning with bandpass filtering as preprocessing. Reported an accuracy of 71.40%.

### 4.2. Comparison of Results

Table 5 summarizes the comparative performance across these studies.

### 4.3. Key Insights and Future Directions

- Feature extraction matters: Studies using advanced feature-extraction techniques, such as differential entropy, achieved significantly higher accuracies. This underscores the importance of incorporating robust features to improve the emotion-classification performance.
- Model architecture: ResNet18 achieved the highest accuracy, showcasing the effectiveness of deep architectures with residual connections in capturing the spatiotemporal dynamics of EEG data.
- Preprocessing: high-performing studies employed artifact rectification and advanced signal processing methods, such as STFT, to enhance the data quality and model performance.
- Limitations of the current study: our models provide a baseline for comparing shallow and deep architectures but lack advanced preprocessing and feature-extraction methods, which likely contributed to the relatively low performance.
- Future work: Future studies should integrate entropy-based feature-extraction techniques, like DE, with architectures such as ResNet or Progressive GCN. Hybrid models combining shallow and deep architectures could further enhance the classification performance while maintaining interpretability.

### 4.4. Conclusions

The comparative analysis revealed that integrating robust feature-extraction techniques and deep learning architectures significantly improved the EEG-based emotion recognition. This study served as a foundation for exploring advanced methods, such as differential entropy and ResNet-based architectures, to bridge the performance gap.

## 5. Discussion

This study explored the use of three different neural network architectures—Shallow FBCSPNet, Deep4Net, and EEGNetv4—for classifying emotions based on EEG signals from the SEED-V dataset. Each model was evaluated under various configurations to determine their effectiveness in distinguishing between five emotional states: happiness, sadness, disgust, neutral, and fear.

### 5.1. Model Analysis

ShallowFBCSPNet: This model, introduced by Schirrmeister et al. [42], was designed explicitly for EEG signal processing by leveraging a combination of band power features extracted from different frequency bands. Among the configurations tested, the best performance was achieved with a learning rate of 0.0001, a weight decay of 0.05, a batch size of 8, and 200 epochs, which resulted in a test accuracy of 39.13%. The model performed well when classifying “Disgust” and “Sad” but struggled with “Fear” and “Neutral”. A second configuration (learning rate = 0.005, weight decay = 0.1) improved the classification of “Happy” and “Neutral” but encountered difficulties with “Disgust” and “Fear”. These results suggest that ShallowFBCSPNet effectively captured frequency-specific features but requires additional tuning or hybrid architectures to generalize across all emotional states.

Deep4Net: The Deep4Net architecture, also described by Schirrmeister et al. [42], is a deeper convolutional network designed to capture complex hierarchical features in EEG data. Its best performance was achieved with a learning rate of 0.01, a weight decay of 0.1, a batch size of 8, and 100 epochs, which yielded a test accuracy of 38.26%. The model excelled in classifying “Sad” and “Happy” but exhibited high misclassification rates for “Fear” and “Disgust”. In a second configuration (learning rate = 0.0005, weight decay = 0.05), the model struggled with similar emotions like “Sad” and “Fear”, reflecting its limitations in separating overlapping features. While the deeper architecture helped capture complex EEG patterns, the results indicate a need for additional training data or regularization to enhance generalization.

EEGNetv4: EEGNetv4, developed by Lawhern et al. [43], is a compact and efficient network designed for EEG-based BCI applications. Despite its lightweight design, the model failed to generalize effectively. In Configuration 1 (learning rate = 0.001, weight decay = 0.1), the model predicted all instances as “Disgust”, indicating severe overfitting. In Configuration 2 (learning rate = 0.01, weight decay = 0.05), all predictions were classified as “Neutral”, reflecting underfitting. With a maximum accuracy of 25.22%, EEGNetv4’s limited depth and reliance on raw EEG data constrained its ability to capture the variability and complexity required for robust emotion classification.

### 5.2. Overall Observations

None of the models consistently performed well across all emotional states, underscoring the challenges of processing raw EEG signals:

- ShallowFBCSPNet: produced the best overall performance but struggled with emotions requiring deeper contextual understanding, such as “Fear” and “Neutral”.
- Deep4Net: captured hierarchical features but struggled with overlapping features in emotions like “Fear” and “Disgust”.
- EEGNetv4: efficient but prone to biased predictions, indicating insufficient feature representation and generalization.

These findings highlight the need for advanced preprocessing, feature extraction, and hybrid model architectures to improve emotion recognition from EEG data.

### 5.3. Challenges and Limitations

The primary challenge observed across all models was their inability to generalize across emotional states, as reflected in the high misclassification rates. For example, EEGNetv4 struggled significantly, with Configuration 1 classifying all instances as “Disgust” and Configuration 2 as “Neutral”, indicating severe overfitting and underfitting, respectively. Although Deep4Net performed relatively well for “Sad” and “Happy”, it struggled to differentiate emotions with overlapping features like “Fear” and “Disgust”. ShallowFBCSPNet, despite achieving the highest accuracy (39.13%), faced difficulties with emotions that required more complex feature extraction, such as “Fear” and “Neutral”.

These challenges stem from the inherent complexity of EEG signals, which are noisy and non-linear, making it difficult for models to separate similar emotional states. Additionally, the SEED-V dataset, while valuable, is relatively small and lacks the variability needed to generalize across diverse emotional expressions. Architectural constraints further compounded these issues: EEGNetv4’s lightweight design was insufficient for capturing complex patterns, while Deep4Net’s depth increased the risk of overfitting with limited data. ShallowFBCSPNet, although effective at extracting frequency-specific features, struggled with capturing the hierarchical patterns required for some emotions. The preprocessing approach also posed limitations. By relying on raw EEG signals with minimal feature engineering, essential information may have been lost, which limited the discriminative power of the models.

## 6. Conclusions

This study evaluated three distinct neural network architectures—ShallowFBCSPNet, Deep4Net, and EEGNetv4—for emotion classification using EEG signals from the SEED-V dataset. The primary objective was to assess their performance in detecting and classifying emotional states while addressing the inherent challenges of EEG data processing. The results demonstrate the potential of these models but also reveal significant limitations in generalization and robustness.

ShallowFBCSPNet achieved the highest accuracy among the models, reaching 39.13%, followed by Deep4Net (38.26%) and EEGNetv4 (25.22%). While ShallowFBCSPNet excelled at classifying emotions like “Disgust” and “Sad”, it struggled with more complex emotional states, such as “Fear” and “Neutral”. Deep4Net performed relatively well for “Sad” and “Happy” but exhibited high misclassification rates for overlapping emotions, like “Disgust” and “Fear”. EEGNetv4, despite its compact and efficient design, suffered from biased predictions, with one configuration predicting all instances as “Disgust” and another predicting all as “Neutral”. These results highlight significant challenges in learning meaningful features from raw EEG signals.

Compared with state-of-the-art studies using the SEED-V dataset, such as Yao et al. [46], which achieved a classification accuracy of 95.61% using ResNet18 and differential entropy (DE), our models underperformed significantly. This underscores the importance of advanced preprocessing and feature-extraction techniques, such as DE, in improving the classification performance. Similarly, studies like Zhou et al.’s [47], which employed Progressive Graph Convolution Networks (PGCNs), achieved 71.40%, further demonstrating the advantages of deeper and more specialized architectures for EEG-based emotion recognition.

The high misclassification rates observed in this study suggest several underlying challenges. First, the inherent complexity of EEG signals, which are noisy and exhibit overlapping features, makes it difficult for models to learn clear distinctions between emotional states. Second, the SEED-V dataset, while valuable, is relatively small and lacks the variability needed to fully capture the diversity of real-world emotional expressions. These constraints likely limited the models’ ability to generalize effectively. Lastly, relying solely on raw EEG signals without advanced feature extraction reduced the discriminative power of the models, especially for subtle emotional differences.

The findings also highlight the differential performance of the models based on their architectures. ShallowFBCSPNet demonstrated strengths in extracting frequency-specific features, making it effective for certain emotions, but struggled with the hierarchical patterns required for emotions like “Fear”. Deep4Net captured more complex features due to its deeper architecture but was prone to overfitting, particularly with the limited dataset. EEGNetv4, while lightweight and computationally efficient, failed to generalize effectively and struggled to learn meaningful features from the data.

These results underscore the ongoing challenges of emotion recognition from EEG signals and the need for advancements in preprocessing, feature extraction, and model design to achieve reliable and robust emotion-classification systems.

### Implications for Future Research

The findings of this study highlight several opportunities for advancing EEG-based emotion recognition. Addressing the challenges observed in this work can significantly improve the robustness and applicability of these systems. Below are key areas for future research:

1. Dataset expansion: The SEED-V dataset, while valuable, has limitations in size and variability. Future studies should focus on collecting larger datasets with a more diverse range of participants and emotional states. Incorporating additional cultural, demographic, and environmental factors would also improve the generalization and applicability across real-world scenarios.

2. Advanced preprocessing and feature extraction: The use of raw EEG signals limited the discriminative power of the models in this study. Employing advanced preprocessing techniques, such as Independent Component Analysis (ICA) and Short-Time Fourier Transform (STFT), could enhance the signal quality by reducing the noise and preserving critical features. Additionally, feature-extraction methods, like differential entropy (DE) or wavelet transforms, could provide more informative representations of EEG data, improving the separation of overlapping emotional states.

3. Hybrid model architectures: Integrating the strengths of shallow and deep architectures could address some of the limitations identified. For instance, hybrid models could combine ShallowFBCSPNet’s frequency-specific feature extraction with Deep4Net’s ability to capture hierarchical patterns, creating more robust and flexible systems. Exploring attention mechanisms could further improve the models’ ability to focus on the most relevant features in EEG signals.

4. Regularization and data augmentation: To mitigate overfitting, future work should explore regularization techniques, such as dropout and weight decay, and employ data augmentation strategies to simulate a broader range of EEG patterns. Methods like adversarial training could also enhance robustness by improving the model performance under noisy or challenging conditions.

By addressing these areas, future research can help overcome the limitations observed in this study and pave the way for more accurate, generalizable, and real-world-ready emotion-recognition systems using EEG signals.

## Figures and Tables

**Table 1:** ShallowFBCSPNet architecture.

Layer (Type)	Output Shape	Param #
Ensure4d	[−1, 62, 10,000, 1]	0
Rearrange	[−1, 1, 10,000, 62]	0
CombinedConv	[−1, 40, 9976, 1]	0
BatchNorm2d	[−1, 40, 9976, 1]	80
Expression	[−1, 40, 9976, 1]	0
AvgPool2d	[−1, 40, 661, 1]	0
Expression	[−1, 40, 661, 1]	0
Dropout	[−1, 40, 661, 1]	0
Conv2d	[−1, 5, 1, 1]	132,205
LogSoftmax	[−1, 5, 1, 1]	0
Expression	[−1, 5]	0

**Table 2:** Deep4Net architecture.

Layer (Type)	Output Shape	Param #
Ensure4d	[−1, 62, 10,000, 1]	0
Rearrange	[−1, 1, 10,000, 62]	0
CombinedConv	[−1, 25, 9991, 1]	0
BatchNorm2d	[−1, 25, 9991, 1]	50
Expression	[−1, 25, 9991, 1]	0
MaxPool2d	[−1, 25, 3330, 1]	0
Expression	[−1, 25, 3330, 1]	0
Dropout	[−1, 25, 3330, 1]	0
Conv2d	[−1, 50, 3321, 1]	12,500
BatchNorm2d	[−1, 50, 3321, 1]	100
Expression	[−1, 50, 3321, 1]	0
MaxPool2d	[−1, 50, 1107, 1]	0
Expression	[−1, 50, 1107, 1]	0
Dropout	[−1, 50, 1107, 1]	0
Conv2d	[−1, 100, 1098, 1]	50,000
BatchNorm2d	[−1, 100, 1098, 1]	200
Expression	[−1, 100, 1098, 1]	0
MaxPool2d	[−1, 100, 366, 1]	0
Expression	[−1, 100, 366, 1]	0
Dropout	[−1, 100, 366, 1]	0
Conv2d	[−1, 200, 357, 1]	200,000
BatchNorm2d	[−1, 200, 357, 1]	400
Expression	[−1, 200, 357, 1]	0
MaxPool2d	[−1, 200, 119, 1]	0
Expression	[−1, 200, 119, 1]	0
Conv2d	[−1, 5, 1, 1]	119,005
LogSoftmax	[−1, 5, 1, 1]	0
Expression	[−1, 5]	0

**Table 3:** EEGNetv4 architecture.

Layer (Type)	Output Shape	Param #
Ensure4d	[−1, 62, 10,000, 1]	0
Rearrange	[−1, 1, 62, 10,000]	0
Conv2d	[−1, 8, 62, 10,001]	512
BatchNorm2d	[−1, 8, 62, 10,001]	16
Conv2dWithConstraint	[−1, 16, 1, 10,001]	992
BatchNorm2d	[−1, 16, 1, 10,001]	32
Expression	[−1, 16, 1, 10,001]	0
AvgPool2d	[−1, 16, 1, 2500]	0
Dropout	[−1, 16, 1, 2500]	0
Conv2d	[−1, 16, 1, 2501]	256
Conv2d	[−1, 16, 1, 2501]	256
BatchNorm2d	[−1, 16, 1, 2501]	32
Expression	[−1, 16, 1, 2501]	0
AvgPool2d	[−1, 16, 1, 312]	0
Dropout	[−1, 16, 1, 312]	0
Conv2d	[−1, 5, 1, 1]	24,965
Rearrange	[−1, 5, 1, 1]	0
Expression	[−1, 5]	0

**Table 4:** Summary of models, configurations, hyperparameters, and performance metrics.

Model	Configuration	Learning Rate	Weight Decay	Batch Size	Epochs	Accuracy (%)	Notes
EEGNetv4	Config 1	0.001	0.1	8	100	25.22	Predicted all as “Disgust”
EEGNetv4	Config 2	0.01	0.05	6	100	24.35	Predicted all as “Neutral”
Deep4Net	Config 1	0.01	0.1	8	100	38.26	Strong for “Sad” and “Happy”
Deep4Net	Config 2	0.0005	0.05	8	100	32.17	Struggled with “Sad” and “Fear”
ShallowFBCSPNet	Config 1	0.0001	0.05	8	200	39.13	Strong for “Disgust” and “Sad”
ShallowFBCSPNet	Config 2	0.005	0.1	4	120	36.52	Better for “Happy” and “Neutral”

**Table 5:** Comparative performance of studies using the SEED-V dataset.

Study	Methodology	Preprocessing	Feature Extraction	Accuracy (%)
This Study	ShallowFBCSPNet, Deep4Net, EEGNetv4	Bandpass filtering, segmentation	None	39.13
Yao et al. (2024) [46]	ResNet18 + DE	Artifact rectification, STFT	Differential entropy (DE)	95.61
Zhou et al. (2023) [47]	Progressive GCN	Bandpass filtering	Learned graph features	71.40

## Data Availability

The data presented in this study are publicly available and can be accessed by submitting a request through the SEED dataset’s official website. Researchers are required to complete a license agreement to obtain access. For further information, please refer to the SEED download page.

## Acronyms

**Acronym**	**Full Term**	**Description**
EEG	Electroencephalography	A method for measuring electrical activity in the brain using electrodes placed on the scalp.
BCI	Brain–computer interface	A technology that enables direct communication between the brain and an external device.
CNN	Convolutional neural network	A type of deep learning model designed for processing data with a grid-like topology, such as EEG signals.
ICA	Independent Component Analysis	A signal-processing method that separates a multivariate signal into additive independent components.
PSD	Power Spectral Density	A measure of a signal’s power content across different frequency components.
SVM	Support Vector Machine	A supervised learning algorithm used for classification and regression tasks.
SEED	SJTU Emotion EEG Dataset	A dataset for emotion recognition from EEG signals is used widely in affective computing research.
SEED-V	SJTU Emotion EEG Dataset (Version V)	An extended version of SEED, with multimodal data and five emotional states.
fMRI	Functional magnetic resonance imaging	A neuroimaging method that measures brain activity by detecting changes in blood flow.
PET	Positron emission tomography	A functional imaging technique used to observe metabolic processes in the body.
T4 GPU	Tensor Core GPU	A type of NVIDIA GPU used for accelerating deep learning tasks.
MNE	MNE-Python	A library for processing and analyzing EEG/MEG signals in Python.
